# Investigation of Environmental Factors as a Key Progression in the Treatment of Fatty Liver Disease: A Study Protocol

**DOI:** 10.7759/cureus.69144

**Published:** 2024-09-10

**Authors:** Nisha D Barole, Vijendra Kirnake

**Affiliations:** 1 Clinical Research, Datta Meghe Institute of Higher Education and Research, Wardha, IND; 2 Gastroenterology, Jawaharlal Nehru Medical College, Datta Meghe Institute of Higher Education and Research, Wardha, IND

**Keywords:** bisphenol a, cirrhosis, gluconeogenesis, perfluoroalkyl substance, socioeconomics

## Abstract

Background

Fatty liver disease (FLD) is currently a global health problem associated with environmental and metabolic diseases. In addition to air pollution, chemicals, and dietary choices, metabolic problems can also contribute to the development of FLD. However, in order to understand this situation, environmental conditions need to be investigated comprehensively.

Materials and methods

This study used a scientific method to assess the environmental factors that play a role in FLD. Individuals from different ethnic backgrounds will be recruited as participants to increase diversity in the sample. The survey will include questions on food, exposure to air pollution, finances, and cultural practices. Statistical analysis will be conducted to further reveal environmental changes and factors that affect FLD, leading to a better understanding of environmental factors that cause FLD in the population.

Results

The study will significantly identify the environmental factors, such as diet, physical activity, exposure to pollutants, etc., that influence the progression and treatment outcomes of FLD.

Conclusion

This study will demonstrate that environmental factors influence the occurrence of FLD.

## Introduction

The liver is considered the most important organ for health because it plays an important role in the metabolism and elimination of toxins. It is particularly necessary for maintaining metabolic balance [1]. Fatty liver disease (FLD) significantly contributes to global mortality and morbidity; effective management is important due to its rising incidence [2]. A number of the liver's biological functions can be changed by things in the environment. The liver's main job is metabolism, including nutrients to energy, synthesizing proteins, and detoxifying harmful substances. These functions are vital for overall health. The liver has a lot of metabolic pathways that are only found there or are very active there. These include gluconeogenesis, the urea cycle, and lipid metabolism [3]. As the number of obese people around the world has grown over the last 30 to 40 years, associated metabolic dysfunction-fatty liver disease (MAFLD) has become a very significant type of chronic liver disease. Metabolic syndrome is characterized by being overweight in the abdominal region, having low high-density lipoprotein (HDL), elevated blood pressure, increased cholesterol levels in the arteries, and/or increased blood sugar [4]. 

Non-alcoholic fatty liver disease (NAFLD) results from things other than drinking too much alcohol. When people drink too much alcohol, they get alcoholic fatty liver disease (AFLD). The liver condition caused by alcohol is one of the main reasons people get chronic liver disease. It is also a main reason for long-term liver disease conditions and one of the main reasons people die from liver problems around the world [5]. Numerous metabolic risk factors are very important in the development of NAFLD, including obesity, insulin resistance, type 2 diabetes, and dyslipidemia. However, there is more and more evidence that some environmental factors may make liver disease more likely. Toxins from landfills are connected to a higher risk of reactive liver disease in the surroundings [6]. In other studies, people with NAFLD consumed more foods that are low in nutrients, high in salt, and high in fat, especially meat-based foods that are high in fat, and fewer fresh fruits [6]. 

Around 25% of people around the world have fatty liver disease (FLD), which can make you develop cirrhosis and liver cancer, which can be fatal. There is a disease called "toxicant-associated steatohepatitis" (TASH) that can happen because of chemicals in the workplace or the environment [7]. Many diseases can affect the liver, and the most common one, FLD, is a result of progress and changes in people's lifestyles. More and more, chemicals, soil and water pollutants, and air pollution are being seen as risk factors that may help the disease start and get worse [8]. Environmental health is a field that is growing, and the body of evidence that connects pollution to human health is always being added to and updated [9].

In the past, researchers have attached FLD to eating poorly, not being active enough, and being around pollutants. There is now more interest among scientists in how outside factors affect the disease and what that means when it starts [10]. External factors include pollutants, industrial chemicals, food contaminants, and socioeconomic factors, among others. These things could be bad for liver health in many different kinds of ways, such as by causing oxidative stress, inflammation, and problems with metabolic pathways [3]. The pollutants found in the air and water can be shown to be harmful to the liver and cause hepatic steatosis. Businesses have linked chemicals like perfluoroalkyl substances (PFAS) and bisphenol A (BPA) to an increase in NAFLD [11]. The rates and outcomes of FLD also vary between groups because of things like differences in income, access to health care, and the quality of the neighborhoods where people live [12].

## Materials and methods

Study design and setting

This observational questionnaire study will be conducted in the Department of Gastroenterology of Acharya Vinoba Bhave Rural Hospital, associated with Datta Meghe Institute of Higher Education and Research (Deemed to be a university), Sawangi, Wardha, Maharashtra, India. The study will be conducted after obtaining consent from the participants diagnosed with fatty liver disease. Permission to proceed with the study was given by the Institutional Ethics Committee Ref. No. DMIHER(DU)/IEC/2024/65, and the study is registered with CTRI (CTRI/2024/04/066475). The study will involve a minimum of 97 subjects who meet the protocol's criteria and will last for one year to collect data. The structured questionnaires that will be used to collect the data [5] (see Appendix 1, Table 4). Table 1 presents the criteria for the inclusion of participants in the study.

**Table 1:** Inclusion and exclusion criteria.

Inclusion criteria	Exclusion criteria
Adults aged 18-65 years	Exclude expecting mothers
No pre-existing liver conditions	Persons who have a history of drinking a lot
Consent for regular follow-ups	Current use of medications influencing liver function

Sample size

The sample size was calculated using a Daniel formula based on the proportion of the estimated population size. The estimation error d = 6.7% and considering the total number of healthcare professionals in the hospital, the population size (n) is 97.

Data collection

Table 2 highlights the tools and procedures used to collect data.

**Table 2:** Data collection methods.

Steps	Procedure	Data collection tool
Participant recruitment	Recruit participants with fatty liver disease through a hospital and obtain informed consent.	Consent form (English, Hindi, Marathi)
Baseline assessment	Will collect demographic information, medical history, and lifestyle factors through questionnaires and personal interviews.	Structured questionnaire
Clinical evaluation	Perform physical examinations and will measure the vital signs. Obtain blood samples for liver and kidney function tests.	Clinical assessment forms
Histological analysis	For those undergoing liver biopsy, collect samples and analyze them for steatosis, lobular inflammation, and hepatocellular ballooning.	Histological analysis reports
Environmental exposure	Administer questionnaires to assess exposure to environmental pollutants.	Environmental exposure questionnaire
Dietary monitoring	Use a food frequency questionnaire and 24-hour dietary recalls to gather dietary data.	Food Frequency Questionnaire
Follow up	Schedule follow-up visits every two months to monitor liver health, environmental exposures, and lifestyle factors.	Follow-up assessment forms
Data management	Enter data into a secure electronic database.	Electronic database system

Primary outcome

The primary outcome of this study is to look into the association between environmental factors (lifestyle, diet, physical activity, and emotional well-being) and the number of people who have fatty liver disease, including individuals residing in Wardha. The liver and kidney function tests inspection are required to detect the disease. Table 3 shows the grading scale for FLD. 

**Table 3:** Grading scale of fatty liver disease.

Grades	Steatosis grade (% of hepatocytes affected)	Lobular inflammation (per 200x field)	Hepatocellular ballooning
Grade 0	<5%	No foci	None
Grade 1	5-33%	<2	Few balloon cells
Grade 2	34-66%	2-4	Many cells/prominent ballooning
Grade 3	>66%	>4	NA

​Statistical analysis

The plan for statistical analysis includes first-level descriptive statistics that show the features of the study population and the way outcome and exposure variables are spread out. Chi-square or Fisher's exact tests will be used for categorical variables, and for continuous variables, t-tests, or ANOVA will be used to look at the connection between each exposure variable and the outcome measures. It will then use multinomial logistic regression analysis to look into the connection between environmental factors, lifestyle choices, exposures, and the number of cases of different NAFLD histological grades, considering things like age, sex, BMI, and other health problems that might make the results less clear. The effects might be different for various groups of people based on things like age, sex, body mass index, and other health problems. The results will also be tested against separate modeling approaches and methods to deal with missing data as part of sensitivity analyses to make sure they are reliable.

## Results

The study will significantly identify the environmental factors (such as diet, physical activity, exposure to pollutants, etc.) that influence the progression and treatment outcomes of FLD. The findings will provide valuable insights into the pathogenesis of FLD and contribute to patient education by highlighting key environmental aspects that patients can manage as part of their treatment regimen.

## Discussion

The primary treatment approach focuses on promoting a healthy lifestyle for both adults and children. This means eating more seafood, poultry, whole grains, nuts, and seeds while consuming fewer ultra-processed foods, fatty meats, sugary drinks, and dishes prepared at a high temperature [13]. Encourage physical activity** **through both informal and structured exercises. This includes everyday activities like walking and gardening and more organized exercise routines such as gym workouts, yoga classes, or sports. It also suggests avoiding smoking and drinking alcohol. Policymakers, community leaders, and school officials need to collaborate to ensure that neighborhoods and schools have safe, walkable areas, culturally appropriate and affordable food options, and safe play areas for kids of all ages [14]. NAFLD worldwide affects 15-30% of people in general. It should be noted that the number of people suffering from NAFLD has increased worldwide, including children and the elderly. According to new data, 25% of adults in the US develop NAFLD each year, while one-third of people in Japan do. In China, the rate of FLD is increasing by 0.594% per year, whereas in Israel, it is rising at a much slower rate of 0.028% per year. 

According to a global meta-analysis, NAFLD affects approximately 25.24% of adults. The most common is the Middle East (31.7%), followed by South America (30.45%), and the lowest is Africa (13.4%). In Iran, 2.9% to 7.1% of the total population suffers from NAFLD or nonalcoholic steatohepatitis (NASH) [15]. Environmental factors, such as endocrine-disrupting drugs (EDCs), have previously been implicated in many diseases, but they might be involved in the formation of NAFLD. It is important to understand the factors that influence the onset and progression of NAFLD. The rise of NAFLD worldwide has become a public health problem, especially in regions where Western-style diets are common. This condition is frequently associated with serious complications such as cirrhosis and hepatocellular carcinoma, and it is more common in children and the elderly compared to middle-aged people [16]. At present period, there has been interest in the effects of environmental toxins on liver health. This is due to the use of many medications, consumption of aflatoxin-free foods, and the presence of cyanobacteria in high-risk areas [3]. The fast growth of trade and industry has led to the release of many chemicals into the environment. Long-term exposure to these pollutants can harm health and affect lipid metabolism [1].

## Conclusions

This study aims to advance the understanding of how environmental factors influence the progression and treatment outcomes of FLD. By assessing a diverse participant sample and examining various lifestyle, dietary, and environmental exposures, we expect to identify key contributors such as diet, physical activity, and pollutants. The study will highlight socioeconomic disparities in FLD prevalence and outcomes, informing the targeted public health interventions and guiding clinical practice. The findings will support the policy development to reduce environmental pollutants and improve urban planning, emphasizing the need for a comprehensive approach that integrates environmental health with clinical care, ultimately serving as a foundation for future studies and interventions.

## Appendices

Appendix 1 

**Table 4:** Structured questionnaires. ALT: alanine transaminase, AST: aspartate aminotransferase, BUN: blood urea nitrogen, GFR: glomerular filtration rate.

Section	Questions	Response
Participant information	Name:	
	Age:	
	Gender:	Male/female/other
	Contact:	
	Date:	
Medical history	Do you have any pre-existing liver conditions?	Yes/No
	Do you have a history of heavy drinking? (more than four drinks on a day or more than 14 drinks per week)	Yes/No
	Do you currently use any medications that influence liver function? Examples include acetaminophen (Tylenol), statins, certain antibiotics (e.g., amoxicillin clavulanate), anti-seizure medications (e.g., valproate), and antifungal drugs (e.g., ketoconazole).	Yes/No
Lifestyle factors	How often do you exercise?	Daily/Weekly/Monthly/Rarely/Never
	What type of exercise do you usually engage in?	Walking/Running/Swimming/Gym/Other
	How would you describe your overall physical activity level?	Very Active/Active/Moderate/Sedentary
	Do you smoke?	Yes/No
	Do you consume alcohol?	Yes/No
Dietary habits	How often do you consume processed foods?	Daily/Weekly/Monthly/Rarely/Never
	How often do you consume high-fat foods?	Daily/Weekly/Monthly/Rarely!/Never
	How often do you consume fresh fruits and vegetables?	Daily/Weekly/Monthly/Rarely/Never
	Do you follow any specific diet?	Vegetarian/Vegan/Low-Carb/Other
	How often do you consume red meat?	Daily/Weekly/Monthly/Rarely/Never
	How often do you consume poultry?	Daily/Weekly/Monthly/Rarely/Never
	How often do you consume seafood?	Daily/Weekly/Monthly/Rarely/Never
	How often do you consume dairy products?	Daily/Weekly/Monthly/Rarely/Never
	How often do you consume whole grains?	Daily/Weekly/Monthly/Rarely/Never
	How often do you consume sugary drinks?	Daily/Weekly/Monthly/Rarely/Never
	How often do you consume snacks and sweets?	Daily/Weekly/Monthly/Rarely/Never
	How often do you consume fast food?	Daily/Weekly/Monthly/Rarely/Never
	Please recall everything you ate and drank in the past 24 hours:	Breakfast, Mid-morning snack, Lunch, Afternoon snack Dinner, Evening snack
Environmental exposure	How often are you exposed to air pollution?	Daily/Weekly/Monthly/Rarely/Never
	What is the primary source of air pollution in your area?	Traffic/Industry/Construction/Other
	Are you exposed to chemicals at work?	Yes/No
	If yes, please specify the type of chemicals:	
	Are you exposed to chemicals at home?	Yes/No
	If yes, please specify the type of chemicals:	
Emotional well-being (PHQ2/9 scale)	Little interest or pleasure in activities related to managing your fatty liver disease, such as following your diet or exercise plan?	Yes/No
	Down, depressed, or hopeless due to your fatty liver disease diagnosis?	Yes/No
	Trouble sleeping or sleeping too much due to worries about your liver condition?	Yes/No
	Poor appetite or overeating because of stress related to your condition?	Yes/No
	Trouble concentrating on health-related tasks?	Yes/No
	Moving or speaking slowly, or being more restless than usual because of your health condition?	Yes/No
	Thoughts that you would be better off dead or of hurting yourself in relation to your liver disease?	Yes/No
Clinical assessment	Blood pressure:	
	Heart rate:	
	Weight:	
	Height:	
	BMl:	
	Liver function test results:	AST, ALT, Bilirubin, Albumin
	Kidney function test results:	Serum creatinine, BUN, GFR
Follow-up	Has there been any change in your medical history since the last visit?	Yes/No
	Have you experienced any new symptoms or health issues?	Yes/No
	Have there been any changes in your lifestyle (diet, physical activity, etc.) or environmental exposure?	Yes/No
Histological analysis	What percentage of hepatocytes are affected by steatosis?	Grade 0: <5%, Grade 1: 5-33%, Grade 2: 34-66%, Grade 3: >66%
	How many foci of lobular inflammation are present per 200x field?	Grade 0: no foci, Grade 1: <2 foci, Grade 2: 2-4 foci, Grade 3: >4 foci
	What is the extent of hepatocellular ballooning?	Grade 0: none, Grade 1: few balloon cells, Grade 2: many cells/prominent ballooning
